# Confounding in Studies on Metacognition: A Preliminary Causal Analysis Framework

**DOI:** 10.3389/fpsyg.2020.01933

**Published:** 2020-08-21

**Authors:** Borysław Paulewicz, Marta Siedlecka, Marcin Koculak

**Affiliations:** ^1^Psychology Department, Faculty in Katowice, SWPS University, Warsaw, Poland; ^2^Consciousness Lab, Institute of Psychology, Jagiellonian University, Kraków, Poland

**Keywords:** metacognition, causal inference, confounding, structural causal model, meta-theory

## Abstract

By definition, metacognitive processes may monitor or regulate various stages of first-order processing. By combining causal analysis with hypotheses expressed by other authors we derive the theoretical and methodological consequences of this special relation between metacognition and the underlying processes. In particular, we prove that because multiple processing stages may be monitored or regulated and because metacognition may form latent feedback loops, (1) without strong additional causal assumptions, typical measures of metacognitive monitoring or regulation are confounded; (2) without strong additional causal assumptions, typical methods of controlling for first-order task performance (i.e., calibration, staircase, including first-order task performance in a regression analysis, or analyzing correct and incorrect trials separately) not only do not deconfound measures of metacognition but may even introduce bias; (3) that the first two problems cannot be solved by using simple models of decision-making derived from Signal Detection Theory. We conclude the paper by advocating robust methods of discovering properties of latent mechanisms.

## 1. Introduction

In this paper, the term metacognition denotes cognitive processes that monitor other cognitive processes, as well as the results of such monitoring, including metacognitive regulation. This broad definition seems to be in agreement with what can be found in the majority of introductory chapters of various monographs on metacognition, of which there are now many (e.g., Nelson and Narens, 1994; Chambres et al., 2002; Koriat and Shitzer-Reichert, 2002; Dunlosky and Metcalfe, 2008; Beran et al., 2012). A monitored or regulated process is sometimes called a first-order process, an object-level process, a type 1 process, or a lower-level process. This naming convention reflects the hierarchical nature of the overall cognitive process responsible for performing tasks involving metacognition. Following this convention from now on, we will use the term “hierarchical task” to denote an arbitrary cognitive task that involves metacognitive monitoring or regulation of any kind.

One of the reasons that it is difficult to study metacognition is that it is a latent mechanism which has a dual causal role, i.e., it monitors and so is influenced by the underlying cognitive process, but, since one of the main functions of monitoring is regulation, it may also regulate and so influence the monitored process. To further complicate the matter, not every case of a first-order process influencing metacognition may represent genuine metacognitive monitoring; for example, a first-order process could become more resource consuming, thus limiting the amount of resources available for metacognition. Similarly, it is theoretically possible for metacognition to influence a first-order process in a non-metacognitive way.

The aim of this paper is to use causal analysis to derive the theoretical and methodological consequences of this special relation between metacognition and the underlying processes. Even though this is a theoretical paper, we made sure that it does not contain any speculative claims: instead of providing our own hypotheses about how metacognition works, we combine causal analysis with the hypotheses expressed by other authors. In that sense, we are proposing a meta-theoretical causal framework for studying hierarchical tasks.

The usefulness of this approach is illustrated by showing how it can help identify important limitations of certain widespread practices in studies on metacognition. We prove that every measure of metacognitive monitoring or regulation is confounded unless strong additional causal assumptions are introduced. In particular, without additional causal assumptions, neither metacognitive judgements (e.g., confidence ratings) nor correlations between performance (e.g., accuracy or sensitivity) and metacognitive judgements are unbiased measures of metacognitive monitoring or regulation. We also show that controlling for first-order task performance may not only fail to deconfound measures of metacognition, but it may even introduce bias. Finally, we show that measures based on Signal Detection Theory or some of its generalizations are just as confounded as simpler measures of statistical dependence because they use the same information in the data. We conclude the paper by advocating robust methods of discovering properties of latent mechanisms.

Almost one-third of our paper is devoted to introducing elements of causal analysis. It is only after we describe the relevant formalism and its interpretation that we begin to address the issues directly related to metacognition. The reason for this is that we cannot assume that a researcher interested in studying metacognition will also be acquainted with causal inference, and we decided not to rely on the introductory books or papers on the subject since they contain much more information than we need to derive the main results. Note also that while we are fairly specific in our criticism of the way in which metacognition is often studied, the constructive part of our paper, in which we try to provide advice on how to do some things better, is rather generic and may not be directly applicable to any specific research problem. This is a consequence of the fact that the problems that we identify are general, but the solutions to these problems depend on the particular characteristics of each study.

### 1.1. Structure and Interpretation of Causal Graphs

We will rely on Pearl's Structural Causal Model (Pearl, 2000; Pearl et al., 2016). At present there is only one alternative theory of causality with similar scope, i.e., Rubin's potential outcomes framework (Rubin, 2005); however, since the two theories are equivalent in the sense that all the axioms of one theory can be derived from the axioms of the other (Galles and Pearl, 1998), the choice is only a matter of convenience. It is impossible to introduce all the major results of SCM in a single paper, so we will describe only the part that seems most readily applicable in typical scenarios when doing basic research.

We will be concerned with qualitative causal structure, i.e., with the issue of the mere presence or absence of causal connections between variables. The quantitative properties of causal relations, such as how to best describe the effect by some deterministic function or statistical model, will be considered only to illustrate a general point. The qualitative structure of causal relations will be represented by graphs consisting of nodes (i.e., variables) and arrows. Unless we state that certain effect simply exists, an arrow from *A* to *B* will represent the assumption that *A* *may* be a direct cause of *B*, which means that the absence of an arrow will represent a stronger assumption (i.e., *A* does not cause *B*) than its presence (i.e., *A* may cause *B*). Here “direct” does not mean immediate, it only means that the effect is not mediated by any other variable in the graph. When arrows represent the mere theoretical possibility of causal effects the graph represents the space of theoretically possible qualitative causal relations. The process represented by an arrow may be arbitrarily complex and multi-staged, but it has to go in the direction of the arrow. In fact, every arrow or node can be thought of as a collapsed graph.

The graph may still be valid even if some of its arrows do not correspond to any real processes, as long as no real arrows connecting the modeled variables are omitted. That is because, as long as they stand for theoretically possible effects, additional arrows may only limit the statistical implications of causal graphs. Moreover, the presence of an arrow from *A* to *B* does not mean that *A* is the only thing causing changes in *B*, and so whenever we draw a graph, unless we clearly state otherwise by saying that some variables are deterministic functions of other variables, we assume that every variable is also influenced by other unspecified factors which can be safely omitted from the graph, except for special cases. In particular, we have to include unspecified factors which may be common causes of variables represented in the graph. Finally, unlike in structural equation models, the effects may be non-linear, and when two or more arrows enter the same node, the joint influence may be interactive.

**Figure 1:**
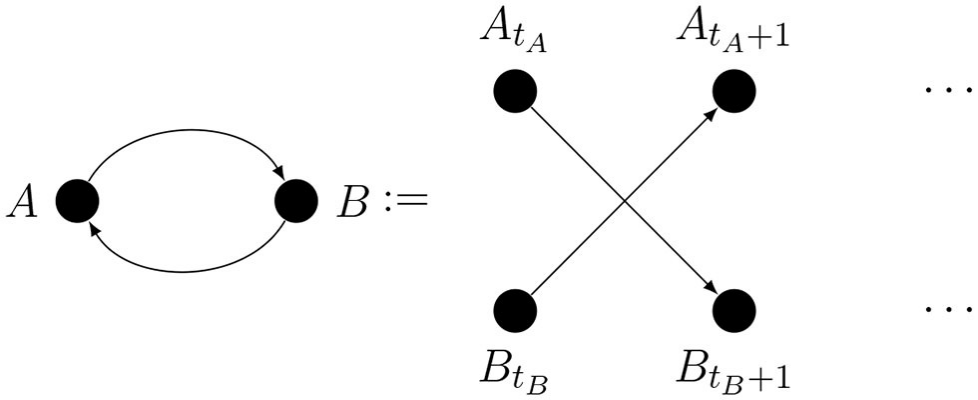
Interpretation of causal loops which represent feedback processes.

Because of the dual causal role of metacognition, there will be causal loops in some of our graphs. Given that causal processes take time, the loop could be taken to mean that causality can go back in time. That is not our intention; A loop may arise because the arrows comprising it cannot be theoretically excluded or because there may be a genuine feedback connection. A real feedback loop can only connect time-aggregated variables and it is shorthand for mutual influence occurring over time, as illustrated by Figure 1.

Here *t*_*i*_ indexes discrete time.

A causal graph can be used to predict, interpret or explain the data because causal relations have statistical implications, e.g., if *A* may cause *B*, then *A* and *B* may be statistically dependent^1^. A path is a finite sequence of adjacent arrows that may change direction along the way, but the sequence must not contain repetitions. The three most important simple cases are the chain (*A* → *B* → *C*), the fork (*A* ← *B* → *C*), and the collider (*A* → *B* ← *C*). The first two paths imply that the outermost variables may be correlated and the correlations due to the given path can be broken by physically fixing the value of the middle variable or by conditioning on the middle variable (e.g., by including the middle variable as a predictor in a regression analysis). The collider represents two independent parent variables that influence a common child; it behaves in an almost opposite way to the other two paths: even if *A* and *C* are independent, they may be *dependent given* *B*, and so regressing *A* on *C* will show no effect; however, regressing *A* on *C* and *B* simultaneously will show the effect of *B* and the spurious effect of *C*. This phenomenon is known as Berkson's paradox (Berkson, 1946) and it shows that introducing additional variables in the regression analysis, e.g., controlling for first-order performance when trying to estimate metacognitive monitoring effects, requires not only statistical but also causal considerations.

We will often make use of the following important fact: variables *X* and *Y* may be correlated according to graph *G* iff there exists at least one collider-free path between *X* and *Y* in *G*. For brevity, we will call such paths conductive. Different conductive paths connecting the same two variables represent alternative but non-exclusive causal explanations of the correlation between the two variables: more than one conductive path between two variables may be true, in which case each path represents a partial explanation of the statistical dependence between these variables.

### 1.2. Identifying Confounding Paths Using Causal Graphs

The causal graph representing a study may contain many variables and many arrows, but usually, the researcher will be primarily interested in only a small subset of paths—often just a single arrow. Following Pearl (2000) we will use the term “target causal quantity” or “target causal effect” to denote any causal relation or causal property of interest. These are impossible to derive from statistical analysis alone because, just as statistical inference requires statistical assumptions, causal inference requires causal assumptions, even in experimental studies. One consequence of this is that, in general, causal questions cannot be answered just by showing that one statistical model fits the data better than another.

The importance of causal assumptions can be illustrated by elaborating on the essential difference between experimental and observational studies. If *X* represents some experimental manipulation and *Y* represents the measured effect, then, given the *causal* assumption of random assignment, the only cause of *X* is a random device, so the only arrow that enters *X* is disconnected from everything else. It follows that there can be no conductive path between *X* and *Y* that enters *X*, hence any conductive path between *X* and *Y* has to leave *X*. Such a path cannot change direction, otherwise it would contain a collider and would not be conductive. Consequently, the observed statistical dependence between any randomly assigned *X* and any measure *Y* can only be explained by the process going from *X* to *Y*: when observed, this statistical relation [i.e., observed *p*(*Y*|*X*)] is an unbiased estimate of the total causal effect of *X* on *Y*. Because of their importance, we will often label randomly assigned variables with the letter *E* as in “experimental manipulation.”

**Figure 2:**
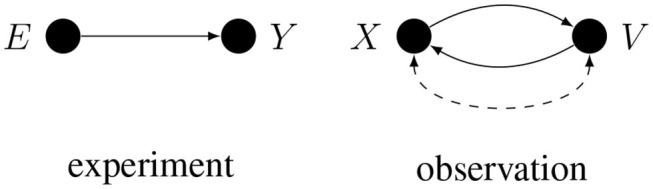
Conductive paths in experimental and observational studies.

Often more than one conductive path corresponds to the same correlation. This is especially true if the two variables are only observed, since in general either one of the two observed variables may cause the other, or the two variables may have common causes, as illustrated in Figure 2.

Here, *E* is randomly assigned, but *X*, *Y*, and *V* are only observed. A dashed arc on the right is shorthand for *X* ← *U* → *V*, where *U* represents all the unidentified common causes of *X* and *V*. When more than one conductive path corresponds to the given correlation, inferring causes from a correlation may require deconfounding, i.e., solving the problem of alternative causal explanations.

In SCM we usually talk about confounding paths, not variables, because a variable by itself cannot imply any statistical dependence. A path is confounding only with respect to some target causal quantity (e.g., *X* → *V*) and its estimate (e.g., correlation between *X* and *V*), just as something is an alternative explanation only with respect to some other explanation and to something that is supposed to be explained. Justifying the preferred causal explanation for the obtained statistical results consists of neutralizing or arguing against the confounding paths (e.g., maybe *V* happens only after *X* has already happened, so *X* ← *V* can be safely deleted). The same goes for the justification of the preferred theoretical interpretation of the chosen measure since validity of measurement is closely related to the relation of being caused by the subject of measurement (Borsboom et al., 2004).

If some plausible alternative causal explanations are not ruled out, i.e., if some confounding paths are not neutralized or broken, then the estimate of the target effect may be biased, i.e., the expected value of the estimate—what it actually measures—may be a mixture of the target causal effect and other confounding effects. In particular, even when the estimated statistical effect is different from zero, the contribution of the target causal effect to the estimate may be null, in which case the researcher will miss the target quantity entirely.

Every confounding path is critical unless something is already known about the relative strength of the relevant causal effects. Unlike noise or measurement error, bias resulting from the presence of confounding paths cannot be dealt with by increasing the sample size because it depends on what is being measured, not on how reliable the measurement is. This bias can only be dealt with—if at all—by changing the design, the method of analysis, or both.

Deconfounding is crucial when doing basic research, especially when the study is concerned with discovering the latent mechanism, such as the mechanism of metacognition. Of course, no study is perfect, but once the confounding paths are identified, they need to be addressed. As is commonly accepted in observational studies, the burden of proof is on the researcher, who omits certain arrows and thus dismisses alternative explanations.

**Figure 3:**
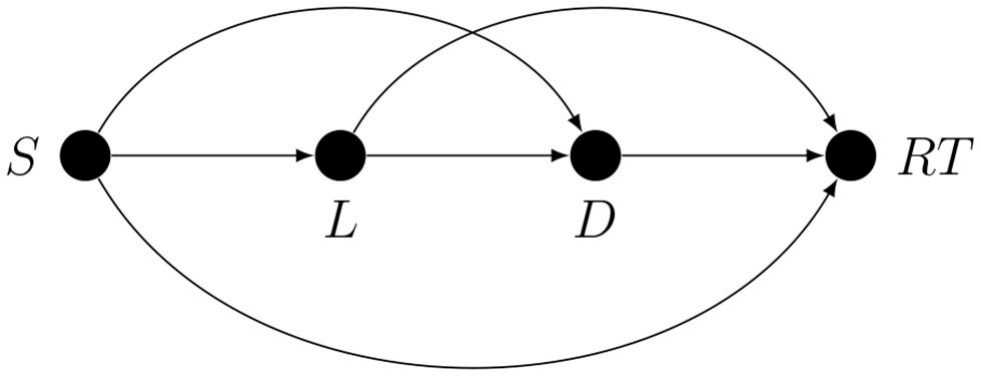
Confounding paths in an experiment with indirect manipulation and measurement.

There are several non-exclusive ways of dealing with confounding. One is by intervention, as in experimental design. However, despite their inherent strength, experimental studies rarely if ever provide definitive answers; this is partly because, especially in disciplines such as psychology, for many variables, it is impossible to alter them directly, and the effects of interest may not be directly observable. For example, let the target quantity be the influence of short-term memory load (*L*) on the duration of the memory search (*D*). *L* is not directly accessible and *D* is not directly observable, and so the set size (*S*) is chosen at random on every trial as a way to indirectly determine the memory load, and recognition reaction time (*RT*) is used as an indirect measure of memory search duration. Thanks to random assignment, the correlation between *S* and *RT* is an unbiased estimate of the total causal effect of *S* on *RT*, but this is not the target quantity. The researcher hopes that the correlation between *S* and *RT* estimates the target quantity *S* → *L* → *D* → *RT*. As an estimate of the target quantity, this correlation may be biased because without additional causal assumptions all that is guaranteed by random assignment of *S* is that the correlation of *S* and *RT* can only be explained by *some* unidirectional path from *S* to *RT*; it does not guarantee that this is the path that the researcher has in mind. In fact, if we assume that the latent effect variable (here *D*) cannot influence the latent cause variable (here *L*), there are exactly three kinds of confounding paths in an arbitrary experiment with indirect manipulation and measurement^2^, as shown in Figure 3.

For example, the *S* → *D* path could represent the metacognitive effect of perceived set size on memory search duration mediated by motivation or effort, not by memory load.

Another approach to deconfounding is by conditioning, i.e., by selecting observations or subjects with some property (e.g., only correct trials), or by introducing additional variables in the possibly non-linear regression analysis (see Pearl et al., 2016, for a more comprehensive treatment). For example, if *X* and *Y* are observed and *Y* cannot possibly influence *X*, then conditioning by regressing *Y* on *X* and all the common causes, if any, without introducing any spurious correlations (by conditioning on a collider) or breaking part of the target path (by conditioning on a mediator or its descendant), would correctly neutralize all the confounding paths. In this case, even though the design is observational, given the causal assumptions it would be possible to estimate *X* → *Y* without bias.

By now it should already be clear why we take the arrow to mean that the causal effect is merely possible. All it takes for some path to provide a valid candidate explanation of the observed correlation is for the path to be theoretically possible and conductive. That is why the fewer arrows there are in the graph, the stronger the assumptions: there are fewer alternative explanations and more can be inferred from data about the generating process. It follows that the more theoretically possible forms of monitoring or regulation there are, the harder it is to deconfound measures of metacognition in general. As we will now show, the relevant literature clearly indicates that it is more difficult to list processing stages that cannot possibly be monitored or regulated than it is to list ones that, at least theoretically, can be.

## 2. A Causal Analysis of a Generic Hierarchical Task

**Figure 4:**
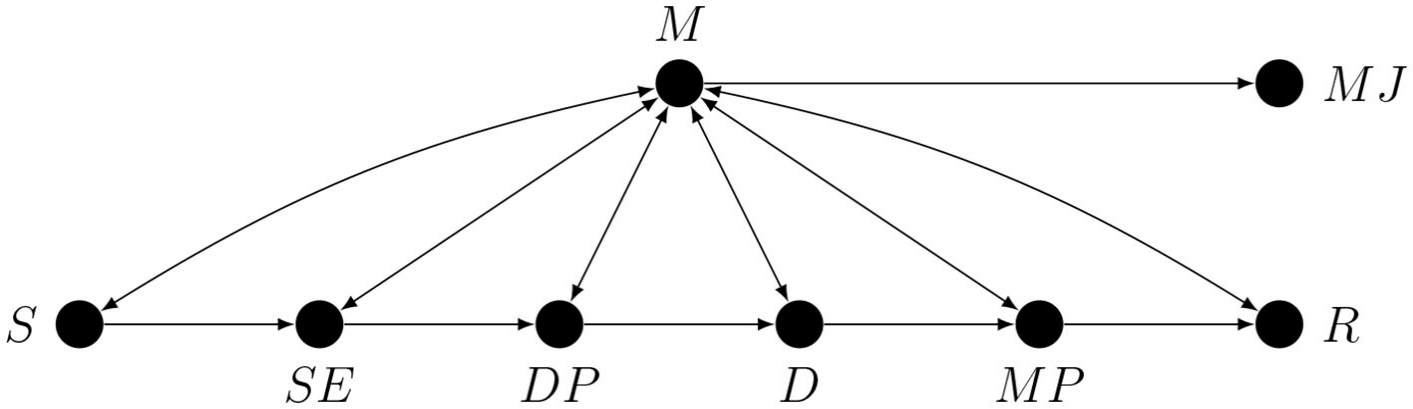
A partial causal graph representing a generic hierarchical task. Here *S* is the stimulus or some stimulus property, *SE* is the stimulus encoding stage, *DP* is the decision process, *D* is the act of making the decision, *MP* is the motor preparation stage, *R* is the response, *M* is metacognition, and *MJ* is the metacognitive judgement.

Metacognition is usually studied using tasks in which the stimuli or their properties can be experimentally controlled and both the first-order (e.g., classification or free recall) and the second-order (some form of metacognitive judgement) responses are measured, sometimes simultaneously. The generic graph representing theoretically possible causal process responsible for performing such tasks is shown later in the paper (see Figure 4). Because of the random assignment of stimulus properties, as a first approximation we can represent such tasks as unidirectional paths going from the stimulus *S* to some first-order response *R*: this is the first-order process. Metacognition can be represented by a node connected with the nodes along the *S* → *R* path.

For our purposes it will be useful to divide the *S* → *R* path into six stages: stimulus *S*, stimulus encoding *SE*, decision- or evidence-accumulation process *DP*, the making of the decision *D*, motor response preparation *MP*, and first-order task response *R*. However, this subdivision into stages is in no way final and only serves as an example of how a preliminary sketch of a causal graph for a given study could look.

We will restrict our attention to monitoring or regulatory processes that operate during a task trial. If the data are not aggregated over trials, then the effects of any trial-level events on subsequent trials can often be safely ignored, which simplifies the graph considerably. This alone is a good reason not to aggregate repeated measures data: for example, by performing statistical analysis on data that are not aggregated over trials, we can ignore the possibility of the alteration of decision criterion used on the following trial caused by the perceived distribution of the stimuli on previous trials, or the possible effect of confidence in a given trial on confidence in the following trials (Rahnev et al., 2015). This is because a path of the form *X*_*i*_ → *Y*_*i*+1_, where *i* is the trial number, cannot connect trial-level variables (i.e., variables with the same *i* value). As far as statistical relations within-trial are concerned, such effects rightly belong to the omitted unspecified factors category, unless some trial-level variable may influence two or more trial-level variables on the following trial, thus forming a conductive path between them.

When deriving the graph for a generic hierarchical task, we will not assume anything about the stimulus or the response other than that the stimulus is randomly assigned. In this way, our model can be applied both to finite alternative forced-choice tasks as well as to tasks where the space of valid responses is not clearly defined, such as learning tasks with a free recall stage.

In the following section, we will provide a non-comprehensive list of theoretical and empirical arguments for introducing specific arrows in the graph that represents the overall process responsible for performing a generic hierarchical task. Note that the fact that we mention a study or a hypothesis does not necessarily mean that we agree with the interpretation of the results given by the authors; The reason that we do not preface most of the interpretations of the results in terms of metacognitive monitoring or regulation with the phrase “according to the authors” is readability. We do not believe that such conclusions are demonstrably false but, as our results imply, establishing the validity of such claims may require careful analysis of confounding paths. Usually, the fact that we list some study as indicating that a certain causal effect may exist only means that the authors expressed a hypothesis that has causal meaning and—because it broadens the scope of possible explanations—that should be taken into account when designing or interpreting the results of an experiment on metacognition.

Some variables that influence metacognition change asynchronously with task stimuli. For example, Samaha et al. (2017) observed that fluctuations in prestimulus alpha-band power are strongly negatively related to confidence ratings, although the relation to accuracy was not detected. Another example is the level of arousal, which fluctuates while a person is doing the task, and may influence cognitive performance on many different levels. Such factors can cause spurious correlations between confidence judgments in different trials, which can be interpreted, for example, as evidence of the influence of confidence in a given trial on confidence in the following trial (see for example: Rahnev et al., 2015). However, because we restrict our attention to processes occurring within trial, here we will ignore such effects.

### 2.1. Some Candidate Metacognitive Monitoring Processes

Most theories of confidence assume that metacognitive assessments are informed by stimulus-related information, such as the quality of a perceptual item, its intensity or its size (e.g., Vickers and Lee, 1998; Galvin et al., 2003; Rhodes and Castel, 2008; Higham et al., 2009), although the degree to which stimuli-related evidence translates to metacognitive assessments varies between theories (see e.g., Kiani et al., 2014; Moran et al., 2015). In some studies, it was found that confidence correlates with experimentally manipulated characteristics of the stimuli, such as presentation time (e.g., Lou et al., 2011), SOA (e.g., Del Cul et al., 2009), and motion coherence/strength (Kiani et al., 2014).

The first-order decision process that follows the stimulus-encoding stage may also be monitored by metacognitive processes. For example, an important class of hypotheses in metamemory studies concerns the relation between fluency or ease of processing and metacognitive judgments (Kelley and Lindsay, 1993; Koriat, 1997; Koriat and Ma'ayan, 2005; Dunlosky and Metcalfe, 2008). Moreover, all the models of choice confidence based on dynamic generalizations of Signal Detection Theory that we are aware of assume that confidence is a function of the history of evidence accumulation (sometimes referred to as “random walk”), such as the drift rate (Ratcliff and Starns, 2009), the distance traversed by the decision-accumulation process scaled by the discriminability parameter (Link and Heath, 1975), post-decisional evidence accumulation (Pleskac and Busemeyer, 2010), or the ratio of smoothed sampled discriminal differences obtained when the response is made (Juslin and Olsson, 1997).

The very act of making a decision may also affect metacognition, for example, by reducing uncertainty (Busemeyer et al., 2006; Kvam et al., 2015; Wang and Busemeyer, 2016; Yearsley and Busemeyer, 2016). Stages of the process of translating the decision to the motor response may also be monitored. Motor response allows action monitoring and control and it seems implausible that the results of performance monitoring (e.g., a failure to execute an intended motor response) would not affect confidence judgments. It has been shown, for example, that response-locked error-related neural activity covaries with confidence level (Scheffers and Coles, 2000; Boldt and Yeung, 2015). A number of studies report correlations between confidence level and action characteristics, such as reaction time (Kelley and Lindsay, 1993; Dougherty et al., 2005; Koriat and Ma'ayan, 2005; Kiani et al., 2014; Fleming et al., 2015; Faivre et al., 2018; Gajdos et al., 2019; Siedlecka et al., 2019; Wokke et al., 2019) or the presence of preparatory motor activity (Gajdos et al., 2019) and such results are typically interpreted as evidence for metacognitive monitoring. Also, the model of self-evaluation proposed by Fleming and Daw (2017) assigns a crucial role to action by assuming that it provides information about one's own decisional process that might not be accessible otherwise.

### 2.2. Some Candidate Metacognitive Regulatory Processes

It seems that the majority of studies on metacognition are concerned with monitoring, while metacognitive regulation is studied less frequently, especially in basic research. Sometimes authors (including us) may even omit the regulatory role when defining the term “metacognition,” stating, for example, that it refers to the ability to monitor one's cognitive processes or to knowledge about ongoing task performance (e.g., Metcalfe and Shimamura, 1994; Fleming and Dolan, 2012; Siedlecka et al., 2016; Fleming and Daw, 2017).

Metacognitive regulation during stimulus-encoding stages is probably ubiquitous, given the assumption that perception is an active process (for review see: Stark and Ellis, 1981; Findlay and Gilchrist, 2001; Henderson, 2003, 2007). The central idea in active perception theories (Gibson, 1966; Bajcsy, 1988) is that behaviors are selected based on the expected information content of the sensory data obtained by those behaviors, and expected information content can be thought of as a metacognitive property because it is relative to current knowledge and to the goals of an agent. A more trivial example of metacognitive regulation of stimulus encoding is the use of mnemonic techniques to improve future memory performance.

The generalizations of Signal Detection Theory provide theoretical arguments for the existence of a regulatory arrow from metacognition to the decision process as well as for the existence of an arrow that enters the stage of making of the decision. According to the common interpretation of the diffusion model (Ratcliff and McKoon, 2008), which is a dynamic generalization of the standard SDT model, the decision process is a kind of noisy evidence accumulation that starts from a possibly biased state and stops when accumulated evidence crosses a decision threshold. There are theoretical and empirical reasons to believe that both the initial bias and the decision thresholds can be metacognitively regulated (Ratcliff and McKoon, 2008). For example, studies on performance monitoring have shown that after encountering a difficulty (e.g., a conflicting stimulus) or after committing an error the subsequent response tends to be slower, which may be an indicator of engaging in a more cautious strategy (Gratton et al., 1992; Ullsperger and Von Cramon, 2001; Veen and Carter, 2006; Dutilh et al., 2012). A similar effect has been shown with real and false accuracy feedback: participants took longer to respond in a trial following negative feedback (Derryberry, 1991; Siedlecka et al., 2020). Finally, Desender et al. (2019) have found that decision bounds that regulate the speed-accuracy tradeoff in the diffusion model are related to the confidence judgement on the preceding trial.

Metacognitive regulation has also been studied in the context of learning. These studies indicate that the allocation of learning time or the selection of learning strategies may be guided by metacognitive monitoring and metacognitive knowledge. For example, feeling-of-knowing judgements positively correlate with the time spent on a question before giving up (e.g., Gruneberg et al., 1977; Reder, 1987, 1988; Nelson et al., 1990; Costermans et al., 1992). Judgements of Learning can be inversely related to self-paced study time, a result which may indicate that the time spent studying an item may depend on monitored or expected changes in the encoding strength (Mazzoni et al., 1990; Mazzoni and Cornoldi, 1993; Dunlosky and Connor, 1997; Thiede and Dunlosky, 1999).

Finally, correlations between motor response properties and confidence judgments found in many studies may also be interpreted as manifestations of the regulatory role of metacognition. For example, the positive correlation between confidence and reaction time may be at least partially explained by the hypothesis that when confidence in a decision is high there is little need to be cautious and the motor execution of the response can be relatively fast (see Gajdos et al., 2019, for a related result).

We are now in a position to draw, in Figure 4 below, a partial causal graph representing the process of performing a generic hierarchical task. We want to stress that this graph is only meant as a simplified illustration of the problem of confounding in studies on metacognition. Note that the fact that there is only one node representing metacognition does not imply that there is only one metacognitive process or module, since, as we have already explained, every node may represent a collapsed graph.

To improve the readability on this graph, causal loops are represented by bi-directional edges. Note that the *S* → *M* arrow represents the possibility of influence of *S* on *M* mediated by stimulus encoding processes unrelated to the first-order task. An immediate consequence of the presence of all the conductive paths in Figure 4 is that without additional strong causal assumptions any single arrow or any path corresponding to a proper subset of conductive paths, such as all the paths involving only monitoring, is, and we cannot stress this enough, *not estimable*.

Because there is more than one possible causal loop in Figure 4 it is hard to say anything in general about the relative importance of various confounding paths, which means that every confounding path is critical. Also note that even if something was already known about the relative strength of various confounding effects making use of this information would not be a trivial task (see for example Pearl, 2012). A researcher who—without accounting for all the possible confounds—claims to have captured for example mostly the *R* → *M* arrow is simply more or less arbitrarily favoring one hypothetical path over other hypothetical paths. Any attempt to justify such a decision on the basis of the results of previous studies will be circular unless the authors of these previous studies have already provided solutions to the relevant confounding problems. To better illustrate the issues involved, we will use the graph from Figure 4 to identify potential confounding paths in studies on metacognitive monitoring.

## 3. Confounding in Studies on Metacognitive Monitoring

The majority of studies on metacognition target metacognitive monitoring. The results of metacognitive monitoring, such as choice confidence, are only observed—they are not experimentally manipulated—and the sources of monitored information are also often not subject to experimental manipulation, at least not directly. Just for this reason, but also because of the possibility of metacognitive regulation, any measure of metacognitive monitoring may be biased.

**Figure 5:**
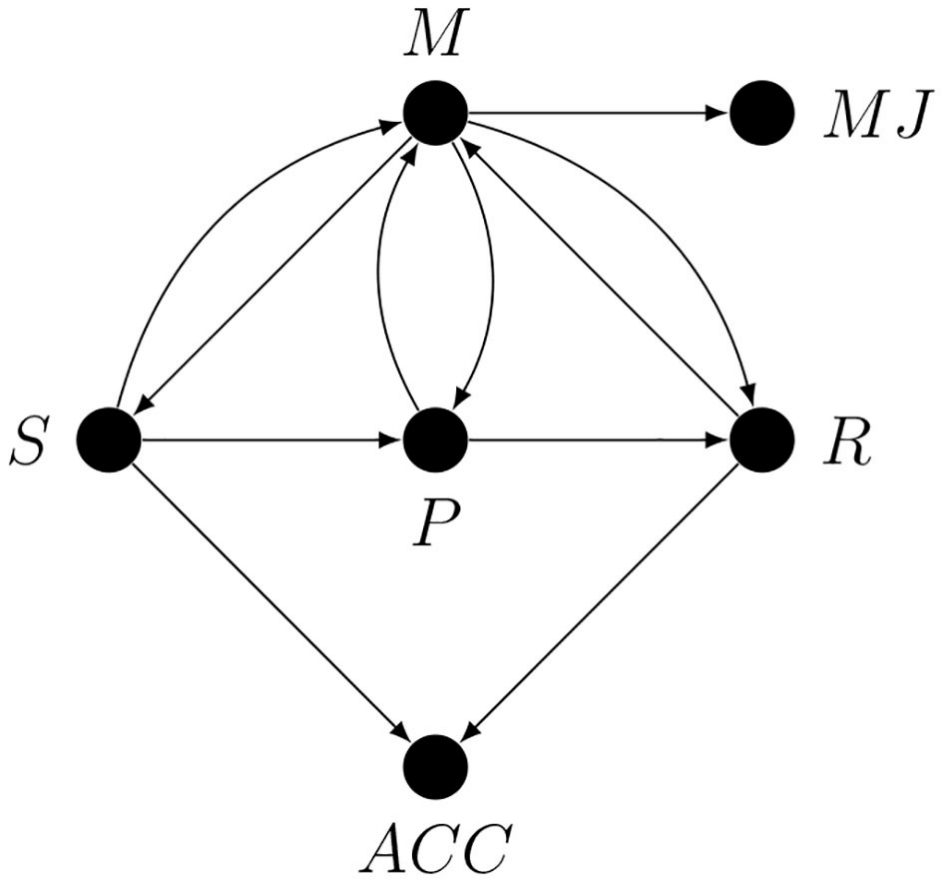
Confounding paths in a generic metacognitive monitoring study.

Imagine that a researcher was interested in metacognitive monitoring, or metacognitive “resolution,” or “accuracy,” but interpreted as a property of metacognitive monitoring. This researcher measured both accuracy and confidence and interpreted their correlation as a measure of metacognitive monitoring. This situation is so common in studies on metacognition that it deserves a graph, shown in Figure 5.

Here *S* is the stimulus, *P* represents all the first-order processing stages between *S* and *R*, *R* is the response, *M* represents all the metacognitive processes, and *MJ* is some metacognitive judgement. The *S* → *M* arrow represents the possibility of monitoring the properties of the stimulus (e.g., if it looks familiar, if it is clearly visible, etc.), *P* → *M* represents the possibility of monitoring arbitrary stages of the first-order decision process, and *R* → *M* represents the possibility of monitoring the first-order response (e.g., if it was quick or slow, or if there was an error in motor execution).

The arrows from *S* and *R* to *ACC* represent the fact that observed accuracy is a deterministic function of the stimulus and the response (*ACC* = 1 iff *S* = *R*). Note that nothing changes in the graph if an estimate of accuracy is replaced by an estimate of sensitivity (i.e., *d*′) since it is also just a deterministic function of *S* and *R*. The arrows from *M* to the first-order processing stages *S*, *P* and *R* represent the assumption that these stages can be metacognitively regulated. For example, *M* → *S* could represent metacognitively guided active perception, and *M* → *R* could represent metacognitively regulated response bias.

If the researcher was interested only in the correlation between *MJ* and *ACC*, then the graph would be irrelevant, but this is unlikely since this correlation by itself has no psychological meaning. If, however, the researcher interpreted this correlation as a measure of monitoring, e.g., of the amount of first-order information available for metacognition, then there are conductive paths that need to be addressed. The only way to justify the claim that this correlation represents metacognitive monitoring is to argue, based on the design of the study and the chosen method of statistical analysis, against the relevance of all the conductive paths between *MJ* and *ACC* that do not correspond to metacognitive monitoring. For example, both the *ACC* ← *S* → *P*_1_ → *M*_1_ → *P*_2_ → *M*_2_⋯*M*_*n*_ → *MJ* path, where *n* is the number of feedback loop iterations, and the *MJ* ← *M* → *P* → *R* → *ACC* path are conductive and connect *MJ* to *ACC*, but the former represents a metacognitive feedback loop involving not only monitoring but also regulation; the latter has nothing to do with metacognitive monitoring, instead, this path represents one way in which metacognitive regulation may contribute to the correlation between *MJ* and *ACC*.

Imagine also that the *MJ*–*ACC* correlation was significantly different in the two experimental conditions, and this difference was interpreted as a measure of the influence of the experimental manipulation on metacognitive monitoring. We could represent this on the graph by introducing the experimental manipulation variable *E* that emits an arrow to *M*. The assumption that the amount of first-order information available for metacognition depends on *E* corresponds to the assumption that the joint influence of *E* and *P* (or *S* or *R*, since these are also stages of first-order processing) on *M* is interactive [e.g., *E*(*M*) = *a*_0_ + *a*_1_*P* + *a*_2_*E* + *a*_3_*PE, a*_3_ ≠ 0, assuming linearity]. This would certainly explain the between-group difference in the *MJ*–*ACC* correlation, but the researcher does not get to choose what is affected by *E*—nature does. If the researcher is interested only in a specific monitoring path such as *P* → *M* or an arrow from a specific stage of *P* to *M*, then this correlation is also confounded with other forms of metacognitive monitoring. Moreover, random assignment of *E* does not change the fact that, given the graph, as a measure of metacognitive monitoring the *MJ*–*ACC* correlation is confounded with metacognitive regulation. Thus, the difference in the *MJ*–*ACC* correlation could also be explained by the effect of experimental manipulation on metacognitive regulation, i.e., interactive effects *E* → *P* ← *M* or *E* → *R* ← *M* (but not *E* → *S* ← *M*, since *S* was randomly assigned).

With some modifications, the graph from Figure 5 can also be used to identify potential confounding paths in studies on the influence of heuristic cues such as fluency, response time, memorizing effort, or familiarity on metacognitive judgement formation. In many such studies, the cues are not directly manipulated, although there are clear exceptions, such as font size, which when directly manipulated correlates with metacognitive judgement (Rhodes and Castel, 2008). The results of such studies are sometimes interpreted as evidence that changes in cues cause changes in metacognitive judgements by informing the monitoring process. However, when the cues are not directly manipulated, the correlation between the cues and metacognitive judgements is not a valid measure of the influence of the cues since there may be common causes of both. Finally, regardless of whether the cues are directly manipulated or not, the correlation between the cues and metacognitive judgments may reflect a complicated process involving iterations of a metacognitive feedback loop.

Common use of simple deconfounding strategies such as controlling for first-order task performance clearly indicates that researchers who study metacognition are well aware of the critical importance of deconfounding. However, as we will now demonstrate, these popular simple deconfounding strategies not only fail to address this issue in its full generality but may even *introduce* bias.

## 4. Why Controlling for First-Order Task Performance May Not Deconfound Measures of Metacognition

A popular approach to deconfounding measures of metacognition, or measures of effects of various manipulations on metacognition, is by attempting to make some chosen performance measure equal between the conditions, either by intervention, as in calibration or staircase^3^, or by statistically controlling for the effect of first-order task performance.

The basic idea, which dates back at least to Nelson (1984), seems simple enough: common sense seems to indicate that if the experimental conditions differ in first-order task performance, then any differences in measures of metacognition can be attributed at least in part to the differences in first-order processing, which makes the latter a confound. If we force the performance measure to be equal in different conditions by calibration, or by using some form of staircase procedure, or if we control for performance in statistical analysis, then, it seems, any remaining differences in metacognition will be deconfounded from the effects of first-order task processing.

Unfortunately, this is not how deconfounding works. Statistically controlling for a variable just because it correlates with the effect of interest may just as easily introduce bias instead of removing it. Trying to intervene on a variable (here by staircasing or calibration) may alter this particular variable and may remove all the other arrows that point to it, but this does not mean that it removes all the confounding paths to which this variable is somehow connected.

In order to achieve deconfounding one first has to consider how confounding may arise: it is only after assuming something about the way in which the observed effects may be causally attributed to first-order and metacognitive processing that something meaningful can be said about the role of controlling for first-order task performance. We will now prove that the claim that controlling for first-order task performance deconfounds measures of metacognition is not true without additional strong causal assumptions and that it is, in fact, unlikely to be true in general. We will only consider two popular ways of controlling for first-order task performance, namely calibration and including the performance estimate in a regression analysis, but with minor modifications, our reasoning can be easily generalized to other cases.

Controlling for first-order task performance by calibration in metacognition studies usually consists of altering the stimuli in the preliminary stage of the experiment in such a way as to make the chosen performance measure more or less equal between the conditions. Anything that we say about calibration can also be said about staircasing, but not vice versa since staircasing is often continued throughout the task. As long as the performance does not change during the experiment, calibration may make any observed differences in measures of metacognition not significantly related to the calibrated performance measure.

Calibration certainly limits the set of possible paths between the stimulus and the response to those that correspond to the fixed performance score. However, this is a purely *quantitative* restriction: it changes the apparent performance of the task, but not how difficult it is since equalization of the targeted performance measure is achieved by introducing the necessary differences in stimulus strength or difficulty. In other words, neither calibration nor staircasing does not solve the problem of equalizing the actual performance, they merely hide it somewhere else. Most importantly, additional causal assumptions are necessary to infer that calibration makes any of the arrows in the graph from Figure 4 disappear. In particular, this procedure is of no help to the researcher who claims that the observed effect is due to any specific arrow, such as metacognitive monitoring of a specific stage along the *S* → *R* path, because without additional causal assumptions all the other monitoring or regulatory arrows are still relevant.

Common trust in the deconfounding power of calibration or staircasing is based on a conceptual error: just because the first-order task *performance measure* was equalized between the conditions does not mean that first-order *processing* was equalized, nor does it mean that *only* first-order processing was affected by calibration. Without additional causal assumptions, it is impossible to say if calibration affects only some first-order processing stages, or if it affects both some first-order stages and some metacognitive processes but does so in a way that makes the performance measure more or less equal between the conditions. In particular, calibration is a stimulus-level intervention and since stimulus properties can be metacognitively monitored, calibration may influence metacognitive monitoring. Because the main function of metacognitive monitoring is to guide metacognitive regulation, calibration may also influence metacognitive regulation. It follows, perhaps surprisingly, that without additional strong causal assumptions calibration deconfounds nothing, all it does is make one statistical effect disappear; With or without calibration or staircase, the difficulty of deconfounding the effects observed for the chosen measure of metacognition remains essentially the same: the researcher either provides theoretical or empirical reasons to believe that all the confounding paths have been addressed or the observed effects cannot be interpreted in terms of any specific path that connects metacognition to the first-order process.

Task performance can also be controlled for statistically. When there is uncertainty as to which performance measure is most relevant, the researcher can perform separate analyses, each time controlling for a different performance measure to see if the results hold. One way to statistically control for first-order task performance is by introducing the performance measure as a predictor in the regression analysis that is aimed at estimating the metacognitive effects of interest. This method succeeds only if (1) it breaks all the confounding paths that are not dealt with by other means and (2) the first-order performance measure is not influenced by any stage along the target path. The second, arguably less obvious but equally important condition is necessary because conditioning on the descendant of a stage along the target path takes away some (or all, if the variable conditioned on *is* a stage along the target path) of the variance due to this path^4^.

**Figure 6:**
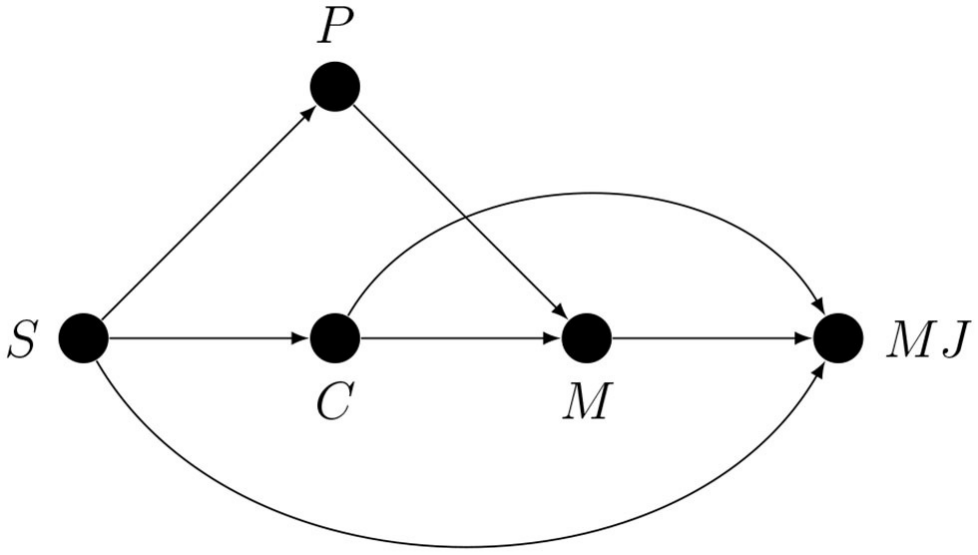
Deconfounding by conditioning on first-order task performance.

To see when conditioning on first-order performance may result in successful deconfounding, consider a study in which some stimulus-level manipulation (*S*) is assumed to influence metacognition (*M*) by affecting some latent cause (*C*). This situation can be represented by a modified graph for an experiment with indirect manipulation and measurement shown in Figure 6. Just for the sake of illustration we assume here that task performance mediates the *S* → *M* confounding path, but the same line of reasoning would apply if it mediated any of the other two confounding paths.

Note that here we generously assume that *P* represents directly observable purely first-order performance, which it never does (there is a measurement error involved which complicates matters even more). As we have explained before, indirect manipulation and measurement allows three types of possible confounding paths. Importantly, the confounding paths may be non-overlapping and it is impossible for the same variable (here *P*) to break (i.e., mediate) more than one distinct path. It immediately follows that without additional causal assumptions which imply that the other two confounding paths can be deleted, deconfounding by conditioning on performance is partial at best, even when the chosen performance measure not only somehow estimates purely first-order processing but also does it without any measurement error. Finally, whenever some form of metacognitive regulation takes place, statistically controlling for first-order performance introduces bias in the estimate of those monitoring or regulatory effects which partially account for the variance in the performance measure.

Our reasoning generalizes to statistical control of first-order task performance when it is built in simplified models of decision-making, such as models of metacognitive judgement based on Signal Detection Theory. In fact, one such model, called meta-*d*′ (Maniscalco and Lau, 2012; Fleming and Lau, 2014), seems to have been explicitly designed to “deconfound metacognition”: according to its authors, one of the meta-*d*′ parameters provides “a bias-free measure of metacognitive sensitivity” that is deconfounded from the effects of first-order performance and bias. We mention these models here to better illustrate the point, but a detailed analysis of their limitations deserves a separate paper. We should stress, however, that our critique applies only to the extent that these models are used to provide estimates of specific components of metacognition —we do not argue against using Signal Detection Theory models in general (quite the contrary, see e.g., Paulewicz and Blaut, 2020).

In every model of metacognitive judgement based on Signal Detection Theory that we are aware of, the process of arriving at a decision is represented either by an internal evidence sample, as in the meta-*d*′ model, or, in the case of dynamic SDT-like models (e.g., Link and Heath, 1975; Juslin and Olsson, 1997; Ratcliff and Starns, 2009; Pleskac and Busemeyer, 2010), by some form of an evidence-accumulation process. Importantly, in these models, the cognitive mechanism is seen as an *abstract* evidence-sampling or evidence-accumulation process and the mechanism by which the evidence was obtained is not explicitly represented. This abstract internal evidence may just as well be the result of purely first-order processing or of an arbitrarily complex interplay of first-order and metacognitive processes.

No part of an SDT model can help in disentangling the vertical arrows in Figure 4, because this model is essentially a measure of statistical dependence between exactly two variables, i.e., the stimulus and the response. The only path that an SDT model—if it is true—can intercept is the one mediated by response bias. In effect, the problem of controlling for purely first-order processing that we have already encountered reappears. To provide deconfounding, an SDT model would have to be extended so that it accounts for additional variables which, when conditioned on, neutralize the confounding paths. However, we are not aware of any such extension of Signal Detection Theory.

## 5. Why Deconfounding Metacognition Is Hard and What Can Be Done About It

As we hope we have already demonstrated, it is not easy to see when successful deconfounding of metacognition is achieved without formal causal analysis, even in the case of widely practiced, intuitively sound and seemingly straightforward control of first-order task performance. The limitations of performance equalization or of fitting simplified models of decision-making as methods of studying metacognition are a consequence of several properties that make metacognition a challenging subject of study: little is known about the mechanism of metacognition, therefore the researcher is forced to consider many arrows and paths, which in turn may force the researcher to address many confounding paths. Moreover, these confounding paths can be particularly problematic because by definition metacognitive processes may be connected uni- or bi-directionally with many different stages of the first-order process.

### 5.1. Robust Approaches to Deconfounding

Ultimately, the limitations of all the approaches to deconfounding metacognition that we have analyzed so far are consequences of strong causal assumptions which are implicit in simplified models or in simple statistical corrections. There are several general-purpose approaches to deconfounding which can be used in studies on metacognition and which are robust in the sense that they may not require strong unsubstantiated causal assumptions. We describe these methods here because compared to a fully-fledged causal analysis targeted at a particular research problem and study design, they are relatively easy to apply, and they may already be familiar to many researchers who study metacognition.

For lack of space, the purpose of this final section of our paper is only to provide a set of pointers and examples of how some already established practices could help in addressing various confounding issues. We want to stress that none of these methods is powerful enough to replace causal analysis. Moreover, their robustness comes at a price: as we have already mentioned at the beginning of our paper, these methods are rather generic, which means that they are not based on strong causal assumptions about the target latent mechanism, and so they may not allow for particularly strong causal conclusions. As we will see, in a way all these methods revolve around the idea of deleting arrows or paths.

#### 5.1.1. Breaking Confounding Paths by Design

Sometimes confounding paths can be guaranteed to be broken because of the design of the study. One example is studies on the effect of response order that some of us were involved in the past (Siedlecka et al., 2016, 2019). These studies were not aimed specifically at deconfounding, but this is irrelevant to the point that we are now making. The main manipulation was the order in which the metacognitive judgement and the first-order response were provided. Although this intervention does not break many confounding paths, when metacognitive ratings are provided first it certainly does break one path, i.e., from the first-order motor response to the metacognitive judgement. In principle, thanks to the simple manipulation of order, such studies are well suited to eventually showing when metacognitive judgement can be influenced by motor response execution.

#### 5.1.2. Deleting Arrows by Arguing for the Null Hypothesis

To the extent that it is possible that the arrows belonging to a confounding path are not real, it makes sense to try to demonstrate this empirically. Interestingly, demonstrating that some conductive path does not exist does not require an unbiased estimate of the path. To see why, imagine that a researcher was interested in the causal effect of *X* on *Y*, but the two variables were only observed and given what is known about them it was also possible that *Y* affects *X*. It follows that the correlation between *X* and *Y* is not an unbiased estimate of either arrow. However, if the researcher managed to demonstrate that *X* and *Y* are statistically independent, then the most likely explanation of this fact would be that neither arrow really exists. The downside is that obtaining evidence of statistical independence is not nearly as straightforward as obtaining evidence of an effect being different from zero.

We are aware of two ways of solving the problem of obtaining evidence for the null, but we will only mention them briefly since this is not a paper on statistical analysis. One popular solution is to use Bayesian inference. The null hypothesis significance testing framework is ill-suited to the task of arguing for the null hypothesis because a lack of statistical significance in no way indicates that the effect does not exist, it only means that it was not reliably detected. Moreover, in frequentist inference, it is impossible to obtain a probabilistic statement about the null hypothesis because in frequentist inference point hypotheses such as a null hypothesis are not points of some probability space, and so frequentist point hypotheses can only be true or false. In Bayesian inference, a set of mutually exclusive and exhaustive hypotheses may form a probability space associated with a prior probability distribution and, once the data are obtained, a posterior probability distribution. A common approach to arguing for the null in Bayesian inference is by using the Bayes Factor in the form of the Savage-Dickey ratio (Wagenmakers et al., 2010). Another solution is to collect enough data points so that the resulting frequentist confidence intervals will be so narrow that if they contain zero it will make sense to say that the effect is either nonexistent or so small as to be negligible. The downside to the Bayes Factor is that it is sensitive to the choice of the prior distribution (Sinharay and Stern, 2002), while the downside to the frequentist approach is that it forces the researcher to justify the choice of the threshold below which the effect size can be considered to be negligible.

#### 5.1.3. Identifying Functionally Distinct Parts of the Latent Mechanism by Selective Modification

Arguing for the null is also an essential part of Sternberg's method of demonstrating separate modifiability by selective influence (Sternberg, 2001), which is a method of process and structure decomposition that has proved useful in the past (see Sternberg, 2001, for examples) and can be reconciled with Pearl's theory of causality. We are aware of five studies on metacognition or consciousness that were interpreted by the authors as demonstrating (partial) selective influence either on performance but not on metacognitive judgement or on metacognitive judgement but not on performance (Lau and Passingham, 2006; Wilimzig et al., 2008; Busey and Arici, 2009; Rounis et al., 2010; Fleming et al., 2015). We should point out that only one of these studies (i.e., Busey and Arici, 2009) contained a discussion of the inherent problems associated with arguing for the null hypothesis; it was also the only study in which the sample size was substantial. In every other case, the authors of the studies claimed—already in the abstract—that one of the effects (either on performance or on confidence) was zero based solely on the fact that it was not significant! Moreover, in some of these studies the effect on confidence was found to be non-significant when conditioning on performance, which is problematic since, as we have already pointed out, first-order task performance may causally depend on metacognitive monitoring and regulation.

Given all of the above, it seems worthwhile to briefly introduce the method of separate modifiability. In its most basic form, this method consists of finding two distinct randomly assigned factors, *F* and *G*, such that (1) given the hypothetical nature of the latent mechanism, *F* and *G* could potentially influence distinct aspects of the mechanism (e.g., stages, processes, or modules), and (2) the effects of each factor are demonstrably independent. The premise is that if there exist functionally distinct parts of a latent process or structure, then it may be possible to selectively influence them, which could be established if there were also distinct measures, each sensitive to one of the distinct parts. It is perhaps worth noting that none of the studies that we have just mentioned demonstrated that two factors selectively influenced two different measures in the same task.

The purpose of separate modifiability is to decompose a latent mechanism by providing information about the separate parts from which it could be composed. By itself, this method does not deconfound anything, but it is a robust method that may help in understanding the problem of confounding by providing information about the latent causal structure.

#### 5.1.4. Abandoning the Idea of Isolating Specific Parts of Metacognition

In order to derive valid conclusions from the study, researchers may have to acknowledge the inherent limitations of the chosen method and settle for a modest interpretation of the results. Similarly, sometimes the only way of dealing with the problem of confounding may be to look for a different target quantity. When deconfounding measures of metacognition, it does not matter if the measure of statistical dependence is theory-based (e.g., meta-d', or SDT thresholds) or not (e.g., logistic regression slope or gamma correlation), because our results hold for *arbitrary* measures of statistical dependence. Nor does it matter if some other variables (e.g., some stimulus property) are randomly assigned: as far as deconfounding is concerned, the only difference between observational and experimental studies is that because of random assignment in experimental studies, some but not all confounding paths can be safely deleted. When there are no good reasons to assume that no metacognitive regulation takes place, researchers can safely interpret such measures only in terms of the *overall* strength of the total, possibly bi-directional, causal connection between some part of metacognition and the first-order process.

This means that often the terms “metacognitive monitoring,” “metacognitive sensitivity,” or “metacognitive efficiency” may have to be replaced with something else. One alternative is to use the term “metacognitive accuracy,” interpreted strictly as denoting the statistical relation between accuracy and some metacognitive judgement; another is to introduce a new term, such as “metacognitive coupling,” to emphasize that some unknown causal connection is there and that it may or may not be bi-directional. Perhaps the term “metacognitive judgement formation,” when used carefully, may also be appropriate. Admittedly, this will often make conclusions much less impressive, but it may also be the only way to ensure that what the researcher argues for is not just wishful thinking, i.e., that the conclusions actually follow from the theoretical assumptions and the data.

## 6. Concluding Remarks

In this paper, we have demonstrated the limitations of common approaches to studying metacognition, including methods specifically aimed at deconfounding. Our analysis shows that detailed questions about metacognition are unlikely to be answered using simple statistical corrections such as conditioning on performance, or by fitting overly simplified mathematical models, such as various generalizations of Signal Detection Theory.

Because by definition metacognitive processes may be connected uni- or bi-directionally with arbitrary stages of first-order processing, confounding is a major problem and formal causal analysis may be required to correctly identify all the theoretically possible alternative causal explanations of the obtained statistical results, or to design a study that can potentially provide unbiased estimates of target causal quantities. It would be unreasonable to expect that every theoretically possible confounding effect has been identified and discussed, but for the causal conclusions to logically follow from the data and the theoretical assumptions, every possible *kind* of confounding effect, i.e., a type of path, such as “other kinds of metacognitive monitoring” or “some kind of metacognitive regulation,” needs to be addressed. The reader who believes that addressing, either directly or indirectly, every plausible alternative causal explanation is too tall an order should be reminded that this is exactly what is commonly required when observational studies are interpreted causally.

As the understanding of metacognition advances, some confounding paths may become irrelevant while new confounding paths may appear, thus making studies that once seemed valid look unconvincing or vice versa. In fact, the theoretical analysis that we have presented in this paper led us to question what we thought our own past studies on metacognition indicated.

We believe that it is not unreasonable to expect that every study provides results which are valid given the explicitly stated assumptions. To this end we have advocated modesty when interpreting the data, using selective influence and special designs that break confounding paths in order to better identify distinct parts of metacognition, and, most importantly, supplementing intuitive understanding of causality with formal analysis.

## Author Contributions

The main results were derived by BP with some help from MS, most of the text was written by BP with great help from MS, with the exception of sections describing the hypotheses about metacognitive monitoring or regulation expressed by other authors, which was written mostly by MS. MK provided valuable feedback and was partially responsible for finding the relevant literature. All authors contributed to the article and approved the submitted version.

## Conflict of Interest

The authors declare that the research was conducted in the absence of any commercial or financial relationships that could be construed as a potential conflict of interest.

## Footnotes

^1^*A* may actually cause *B* and yet the two variables may be statistically independent, but this happens in special cases that can be ignored most of the time.

^2^This can be proved: if *S* is randomly assigned, then the correlation between *S* and *RT* is an unbiased estimate of the unidirectional path from *S* to *RT*. Any unidirectional path from *S* to *RT* has to leave *S* and it either enters *L* or not. If it does enter *L*, it either enters *D* or not. From *D* it can only go to *RT*. If it does not enter *D*, it has to go to *RT* directly. If it does not enter *L*, it either enters *D* or not. If it does not enter *D*, it has to go to *Y* directly. Here we are assuming that *L* and *D* are not time-aggregated variables that can hide mutual influence, otherwise there are additional possible confounding paths.

^3^The limitations of staircasing in studies on metacognition were recently discussed by Rahnev and Fleming (2019) who claimed that by introducing variability in stimulus strength, staircasing may have an undesirable effect on estimates of metacognitive effects. In the paper, the authors consider this to be an important finding for which they even introduce a new term (i.e., “metacognitive inflation”). However, although we also believe that staircasing may sometimes lead to estimate bias in studies on metacognition we do not endorse the authors' reasoning. Firstly, Rahnev and Fleming's claim is causal (they use the terms “influence” or “affect” to describe it) but this claim is based on observational data; the authors re-analyzed some available datasets by selecting the trials with constant or variable stimulus strength, i.e., stimulus strength variability was *not* randomly assigned. Secondly, in our view the authors should examine to what extent their results can be explained by the simple *statistical* fact that—by definition—non-linear estimates (in this case the area under the ROC 2 curve and the meta-*d*′ based measures) may be asymptotically biased when based on data aggregated over different values of a correlated variable (in this case the stimulus strength).

^4^This may also introduce bias in another way (see Pearl, 2012).
